# Potential Immune Indicators for Predicting the Prognosis of COVID-19 and Trauma: Similarities and Disparities

**DOI:** 10.3389/fimmu.2021.785946

**Published:** 2022-01-20

**Authors:** Hamed Fouladseresht, Atefe Ghamar Talepoor, Nahid Eskandari, Marzieh Norouzian, Behrooz Ghezelbash, Mohammad Reza Beyranvand, Seyed Aria Nejadghaderi, Kristin Carson-Chahhoud, Ali-Asghar Kolahi, Saeid Safiri

**Affiliations:** ^1^ Department of Immunology, School of Medicine, Isfahan University of Medical Sciences, Isfahan, Iran; ^2^ Department of Immunology, School of Medicine, Shiraz University of Medical Sciences, Shiraz, Iran; ^3^ Department of Laboratory Sciences, School of Allied Medical Sciences, Hormozgan University of Medical Sciences, Bandar Abbas, Iran; ^4^ Social Determinants of Health Research Center, Shahid Beheshti University of Medical Sciences, Tehran, Iran; ^5^ Research Center for Integrative Medicine in Aging, Aging Research Institute, Tabriz University of Medical Sciences, Tabriz, Iran; ^6^ Systematic Review and Meta-Analysis Expert Group (SRMEG), Universal Scientific Education and Research Network (USERN), Tehran, Iran; ^7^ Australian Centre for Precision Health, Allied Health and Human Performance, University of South Australia, Adelaide, SA, Australia; ^8^ School of Medicine, The University of Adelaide, Adelaide, SA, Australia; ^9^ Social Determinants of Health Research Center, Department of Community Medicine, Faculty of Medicine, Tabriz University of Medical Sciences, Tabriz, Iran; ^10^ Immunology Research Center, Tabriz University of Medical Sciences, Tabriz, Iran

**Keywords:** COVID-19, cytokine, prognosis, T cell, trauma

## Abstract

Although cellular and molecular mediators of the immune system have the potential to be prognostic indicators of disease outcomes, temporal interference between diseases might affect the immune mediators, and make them difficult to predict disease complications. Today one of the most important challenges is predicting the prognosis of COVID-19 in the context of other inflammatory diseases such as traumatic injuries. Many diseases with inflammatory properties are usually polyphasic and the kinetics of inflammatory mediators in various inflammatory diseases might be different. To find the most appropriate evaluation time of immune mediators to accurately predict COVID-19 prognosis in the trauma environment, researchers must investigate and compare cellular and molecular alterations based on their kinetics after the start of COVID-19 symptoms and traumatic injuries. The current review aimed to investigate the similarities and differences of common inflammatory mediators (C-reactive protein, procalcitonin, ferritin, and serum amyloid A), cytokine/chemokine levels (IFNs, IL-1, IL-6, TNF-α, IL-10, and IL-4), and immune cell subtypes (neutrophil, monocyte, Th1, Th2, Th17, Treg and CTL) based on the kinetics between patients with COVID-19 and trauma. The mediators may help us to accurately predict the severity of COVID-19 complications and follow up subsequent clinical interventions. These findings could potentially help in a better understanding of COVID-19 and trauma pathogenesis.

## 1 Introduction

Inflammation is a complex cascade playing a dual role in both physiological and pathological conditions. Inflammatory responses restrict infections and induce tissue repairing by the local release of different immune mediators and recruitment of immune cells (1). However, if the process becomes uncontrolled and systematic, it can be destructive and cause multiple organ failure (MOF) (2). The latter is evident in patients with severe coronavirus-2019 (COVID-19) and severe trauma (2, 3).

COVID-19 is a viral disease caused by severe acute respiratory syndrome-coronavirus (SARS-CoV)-2. COVID-19 can begin with either slight or substantial changes in circulating immune cell distributions and/or functions, followed by cytokine storm (CS), which can ultimately result in MOF (4). The fallout from a CS is a rapid increase in circulating levels of pro-inflammatory cytokines including interleukin (IL)-6, IL-1, tumor necrosis factor (TNF-α), and interferons (IFNs). The CS has a damaging effect on human organs by impacting the transition of various immune cells, such as macrophages, neutrophils, and T cells, into various tissues (4). COVID-19 presents with a broad spectrum of clinical symptoms (5). Diagnosis is typically confirmed by chest computerized tomography (CT) scans and real time-quantitative polymerase chain reaction (RT-qPCR) (6, 7). Laboratory findings, including complete blood count (CBC), blood levels of inflammatory mediators, and coagulation factors, can further predict and monitor COVID-19 complications (8).

Similarly, trauma is a polyphasic inflammatory condition, which in severe form induces complex host immune responses, disrupts immune system homeostasis, and predisposes patients to opportunistic infections and inflammatory complications (9). After severe injuries, large amounts of mediators called damage-associated molecular patterns (DAMPs) are released into the circulation triggering the innate and adaptive immune responses (10). The recognition of DAMPs by immune cells induces systemic inflammatory response syndrome (SIRS) that finally result in physiological changes like hypo or hyperthermia, increased heart rate, leukocytosis, lymphopenia, thrombocytopenia, and MOFs (11). Subsequently, to restrict the excessive pro-inflammatory response, the long-lasting compensatory anti-inflammatory response syndrome (CARS) is evoked and caused post-traumatic immunosuppression (IS) (12). Initially, both CARS and IS suppress trauma-induced inflammation and promote a natural healing response to control immune reactivity to tissue damage and restore immune system homeostasis (13). Conversely, persistent both situations (CARS and IS) can suppress adequate antimicrobial immunity resulting in increased susceptibility to opportunistic infections and serious complications like sepsis and septic shock with following organ failure (14). Sepsis is a major leading cause of mortality and morbidity in trauma patients. Indeed, it is difficult to detect the timing of sepsis in trauma patients because severely injured patients usually present with SIRS (15).

In the setting of trauma, although traumatic insult is considered as the first driver of inflammatory responses, other hyperinflammatory states, such as COVID-19, has also the capacity to augment the inflammation (16, 17). Recent evidence reported elevated inflammatory response in COVID-19 patients sustaining orthopaedic trauma injuries due to their baseline hyperinflammatory states (16). Moreover, it has been documented that the clinical characteristics of COVID-19 patients with fractures were more serious than those of patients without fractures (18). Other studies also found higher intensive care unit (ICU) admission and mortality rates after elective surgery on asymptomatic COVID-19 patients (18, 19). A retrospective study indicated higher mortality and complications rates in patients with active COVID-19 who were over 70 years of age with orthopaedic trauma surgery (20). Furthermore, study of patients admitted to Pennsylvania trauma centers showed that traumatic injury concomitant with COVID-19 infection may increase risks of morbidity and mortality (21).

Even though many immune mediators are affected by the inflammatory condition of trauma, complicating their ability as an outcome predictor in COVID-19 (22), testing immune mediators can still be a rapid and inexpensive method of predicting outcomes for COVID-19 infections (23). Therefore, there is an urgent need to investigate and compare the kinetics of immune mediators after the onset of COVID-19 symptoms and traumatic injuries, to identify the most appropriate mediators and evaluation times to more accurately predict complications in COVID-19.

The current review aimed to investigate similarities and differences in common inflammatory mediators, cytokine/chemokine levels, and immune cell subtypes, based on the kinetics between patients with COVID-19 and trauma. Particularly as there is potential for each variable to become a target in the prognosis of COVID-19 within the trauma context.

## 2 Methods

Published articles for inclusion in this evidence synthesis were identified through a PubMed database search undertaken on August 3, 2021. No search filters or limits were used on publication type, language, time period, or any other fields. The Medical Subject Heading (MeSH) terms searched included ‘Coronavirus Infection’ or ‘Coronavirus’ or ‘SARS-CoV-2’ or ‘COVID-19’ or ‘2019-nCoV’ AND ‘Cytokines’ or ‘C-reactive protein’ or ‘Procalcitonin’ or ‘Ferritins’ or ‘Serum Amyloid A Protein’ or ‘Interleukins’ or ‘Interleukin-1’ or ‘Interleukin-6’ or ‘Tumor Necrosis Factor-alpha’ or ‘Interleukin-4’ or ‘Interleukin-10’ or ‘Leukocyte Count’ or ‘Neutrophils’ or ‘Monocytes’ or ‘Lymphocytes’ or ‘Lymphocyte subsets’ or ‘T-Lymphocytes, Helper-Inducer’ or ‘Th1 Cells’ or ‘Th2 Cells’ or ‘Th17 Cells’ or ‘T-Lymphocytes, Regulatory’ or ‘Tr1 Cells’ or ‘Suppressor T-Lymphocytes, Naturally-Occurring’ or ‘T-Lymphocytes, Cytotoxic’ or ‘TC1 Cells’ or ‘TC2 Cells’. Grey literature searching involved a search of the medRxiv website (https://www.medrxiv.org/) to identify pre-print articles. Manual screening of reference lists of all relevant publications was conducted to identify further qualified studies.

## 3 Kinetics of Immunological Mediators in Patients With COVID-19 and Trauma

### 3.1 Common Inflammatory Mediators

During infection and trauma, the secretion of pro-inflammatory cytokines including IL-1, IL-6, and TNF-α, induce the production of C-reactive protein (CRP), procalcitonin (PCT), ferritin, and serum amyloid A (SAA) as common inflammatory mediators. Several studies have shown that some common inflammatory mediators might be linked with severity of complications after COVID-19 and/or trauma (24–26). Therefore, it’s likely that serum levels of common inflammatory mediators may be a valuable candidate in the prognosis of COVID-19 and trauma, as discussed below.

#### 3.1.1 CRP

CRP is an acute-phase protein secreted by hepatocytes in the presence of pro-inflammatory cytokines (27). Under steady-state, the blood concentration of this acute-phase protein is less than 10 mg/L. In the presence of an infection, levels increase within 6-8 hours, peak at around 24-48 hours post-infection, and then rapidly return to normal levels within 4-9 hours (28). The relatively low levels of CRP during viral infections compared to bacterial infections may be described by the inhibitory role of IFN-α (29).

COVID-19 patients with a higher serum level of CRP at admission (3-5 days post-SARS-CoV-2 infection when symptoms appear), which continues for 2-3 weeks, have an additional risk for poor prognosis. Whereas a decrease in serum level of this protein on the third week, following a gentle increase during the first and second weeks post-symptom onset, correlates with good disease outcomes (30). Higher CRP levels at admission have been reported in COVID-19 patients with more severe symptoms (24, 31). Also, a positive correlation between serum levels of it at admission and chest CT progression has been detected in recent studies (24, 32–34). Han et al. have indicated the optimal cut-off point of CRP at admission is 3.38 mg/L, which may be applicable to predict COVID-19 outcomes (33).

Since patients with trauma are referred to trauma centers approximately 1-4 hours after injury, CRP levels at admission have not correlated with poor outcomes post-trauma (35, 36). It typically increases within 6-12 hours, reaching a peak after 24-72 hours (37). This increase is on days 1 to 3 after admission, which continues for the following days, and is associated with poor outcomes (38). Whereas a decrease in this protein after a temporary increase at the time of admission is correlated with good prognosis (38). Previous studies have shown a correlation of CRP serum levels within 24 to 72 hour period post-trauma with severe infectious complications (37, 39). Therefore, a cut-off point ≥154.4 mg/L of during 24-48 hours post-trauma, could help predict post-trauma infection complications (39).

#### 3.1.2 PCT

PCT is a 16-amino acid peptide that is normally produced by thyroid parafollicular cells and is released by mucosal neuroendocrine cells in response to pro-inflammatory cytokines. The normal serum level of it in healthy individuals is less than 0.05 ng/mL. In inflammatory conditions it begins to increase within 4 to 12 hours, reaching up to a 1000-fold increase within 12 to 24 hours, with a decrease after 24 hours. Secondary elevation of PCT might be observed after 72 hours, depending on the severity of inflammation (40, 41). The short half-life of procalcitonin (24-30 hours) in peripheral blood makes it an ideal candidate to predict the prognosis of inflammatory disease outcomes like bacterial infections (42).

Several studies have clarified that in viral infections, such as SARS-CoV-2, the mean serum level of PCT is <0.5 μg/L (43–45). However, 15-25% of COVID-19 patients with poor outcomes have a mean serum PCT level ≥0.5 μg/L at admission (7, 46, 47). A positive correlation has been reported between serum levels of this protein at admission and severity of COVID-19 (48). The serum levels >0.5 µg/L at admission might be considered as an optimal cut-off value for the assessment of adverse outcomes (49, 50). Kinetically, serum PCT levels >0.5 µg/L at admission that continue until days 9-11 are associated with secondary elevation of it and a 5-fold increased risk of critical bacterial co-infection for COVID-19 patients. It appears that the relationship between elevated PCT sera and poor prognosis in COVID-19 is due to common bacterial co-infections (7, 46, 47).

Increased serum levels of PCT have also been reported as a poor prognostic indicator in trauma patients in at least two-time points (51, 52). The significance of the first elevation of this protein within 4 to 48 hours of trauma, is related to the magnitude of tissue injury and is a poor prognostic indicator for early complications such as SIRS, CARS, and persistent inflammation, immunosuppression, and catabolism syndrome (PICS). There is no significant increase during the period of 4 to 48 hours after mild trauma (52, 53). The second elevation of serum PCT levels within 3 to 7 days post-injury can be attributed to opportunistic bacterial infections and sepsis due to a compromised immune system and early CARS and PICS in severe trauma. Infection-induced SIRS, CARS, and PICS have stronger correlations with poor prognostic outcomes of trauma compared to trauma-induced types (38, 54, 55). These results have been observed in both survivor and non-survivors cases following traumatic injuries, respectively (56).

#### 3.1.3 Ferritin

Ferritin is a 480-kD iron storage protein in hepatocytes and reticuloendothelial cells, which has a very low level in blood circulation. Ferritin plays an important role in cellular antioxidant defense mechanisms by sequestering free cytosolic iron rapidly (57). Increased ferritin serum level is an indicator of many inflammatory conditions, including acute infections and injuries (58). It is also the hallmark of macrophage activation syndrome, adult-onset Still’s disease, and septic shock (59, 60). Possible mechanisms increasing this protein in serum, include 1) A consequence of cell lysis, which ferritin releases from intracellular storages; 2) A reflection of inflammatory response intensity, where ferritin is produced under the effect of inflammatory cytokines such as IL-6, IL-8 and TNF-α (57, 61); and 3) A protective response in oxidative stress to sequestrate free iron (62).

One study evaluating ferritin serum levels on admission showed an increase of this protein in patients with non-severe COVID-19, within a normal range (63), however, another study identified an abnormal elevation of serum ferritin among patients with critical-to-severe COVID-19 (57). Elevation of serum ferritin level starts on the first day after infection (57) and could be considered as an indicator of poor COVID-19 prognosis. Two independent studies reported that serum levels of ferritin and IL-6 are increased in patients with severe COVID-19 and decreased in recovered cases (45, 64). These studies suggest that macrophage activation along with increased serum levels of inflammatory cytokines such as IL-6 might be contributing to an increasing ferritin serum level in COVID-19 patients. Ferritin, in turn, might promote oxidative stress, the secretion of IL-1β and IL-10, as well as, macrophage activity (57).

Similar to COVID-19, serum ferritin level at admission may be a predictor of trauma outcomes. Some studies have reported that serum levels of this storage protein depend on trauma severity, as assessed by the injury severity score (ISS) (65, 66). High serum levels of ferritin at admission are associated with poor prognosis in patients with trauma (66). Sharkey et al. showed that a serum level >270 ng/ml of ferritin for women and a level >680 ng/ml for men at admission are associated with the development of progressive complications in trauma patients (66). Meanwhile, results from another study indicated a positive correlation between serum ferritin levels during days 1 to 2 post-trauma with poor prognosis in ICU (65–68).

#### 3.1.4 SAA

SSA is a multifunctional protein involved in metabolic and immunological responses and is produced as an acute-phase protein during inflammation (69). Under inflammatory conditions, SAA is changed in kinetic patterns similar to PCT. In normal level, it binds to high-density lipoproteins (HDL) to recycle cholesterol in the cell membranes and repair damaged tissue, whereas high concentrations of SAA promote gene expression of inflammatory cytokines, immune cell recruitment, low-density lipoproteins (LDL) oxidation, reactive oxygen species (ROS) generation, and the survival time of neutrophils (70, 71). Moreover, SAA is a precursor of amyloid A protein (AA), an insoluble and fibrillar protein, which causes secondary amyloidosis and increases the risk of organ failure and early death (72). Overall, high rates of SAA stimulate the inflammatory process in inflammatory conditions and might be a poor prognostic sign for inflammatory diseases.

Studies have identified significantly higher concentrations of SAA at the time of admission in patients with severe COVID-19, compared with healthy controls (30, 73, 74). A study by Fu et al. revealed that serum levels>157.9 mg/L of this protein at admission can be an appropriate cut-off point to predict the severity of COVID−19 (73). Thus, the level of the SAA at admission is associated with the incidence of poor outcomes in COVID-19 patients (30, 73, 74).

Serum levels of Amyloid A (AA) during 6 to 24 hours post-trauma have been suggested as a prognostic indicator to predict poor outcomes in patients (75, 76). Another study showed a positive association between severity of injuries and increased AA concentrations in blood circulation after trauma (77). Most data about the association between SAA levels and severity of injuries are related to traumatic brain injury (TBI) patients (78, 79). Additionally, Carabias et al. have shown that both patients with moderate-to-severe and mild TBI have higher SAA levels compared to healthy controls (75).

**Figure 1:**
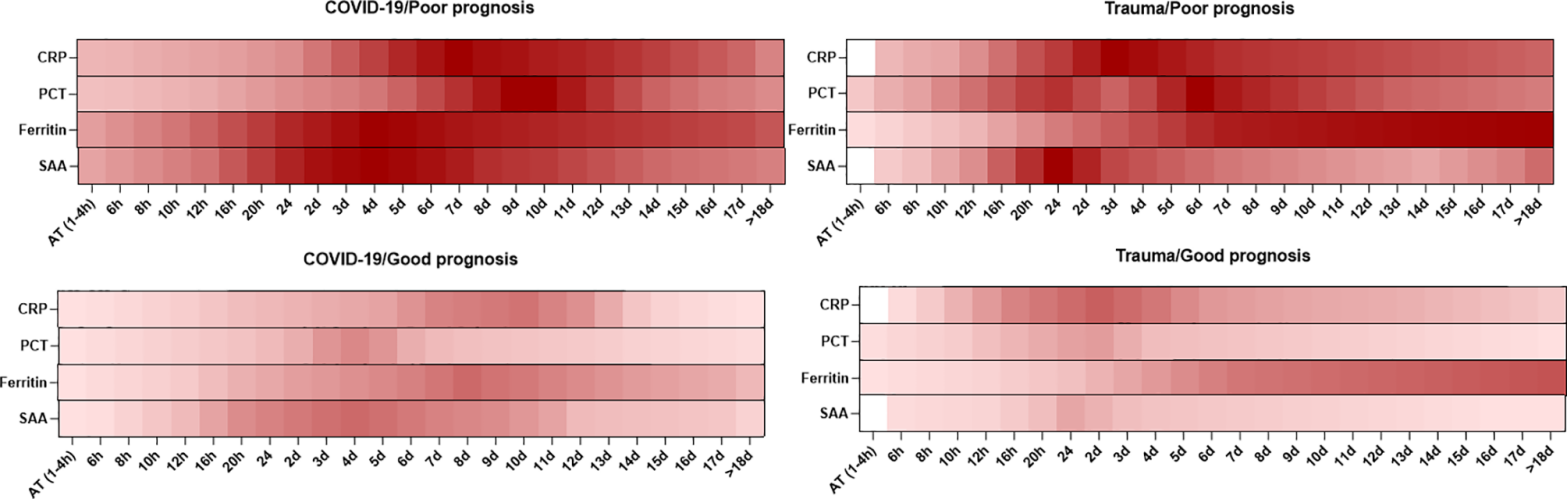
Association between the kinetics of changes in serum levels of inflammatory mediators with prognosis in patients with COVID-19 and trauma. The heat map represents the kinetics of changes in serum CRP (30, 32, 33, 37, 80), PCT (30, 33, 38, 51–53, 55), ferritin (33, 68) and SAA (73, 75, 76, 78) levels during 18 days after admission in COVID-19 and trauma patients associated with poor and good prognoses. COVID-19 patients with an increased risk of mortality, MOF, severe-to-critical forms of the disease, intensive care unit admission, and/or hospitalization are defined as a poor prognosis for the disease. Conversely, patients with the opposite are defined as having a good prognosis for the COVID-19. AT, admission time; CRP, c-reactive protein; d, day; h, hour; MOF, multiple organ failure; PCT, procalcitonin; SAA, serum amyloid A.

Overall, the dynamic changes in serum levels of inflammatory mediators are shown to have an association with prognosis in patients with COVID-19 and trauma (**Figure 1**).

### 3.2 Cytokines/Chemokines

**Figure 2:**
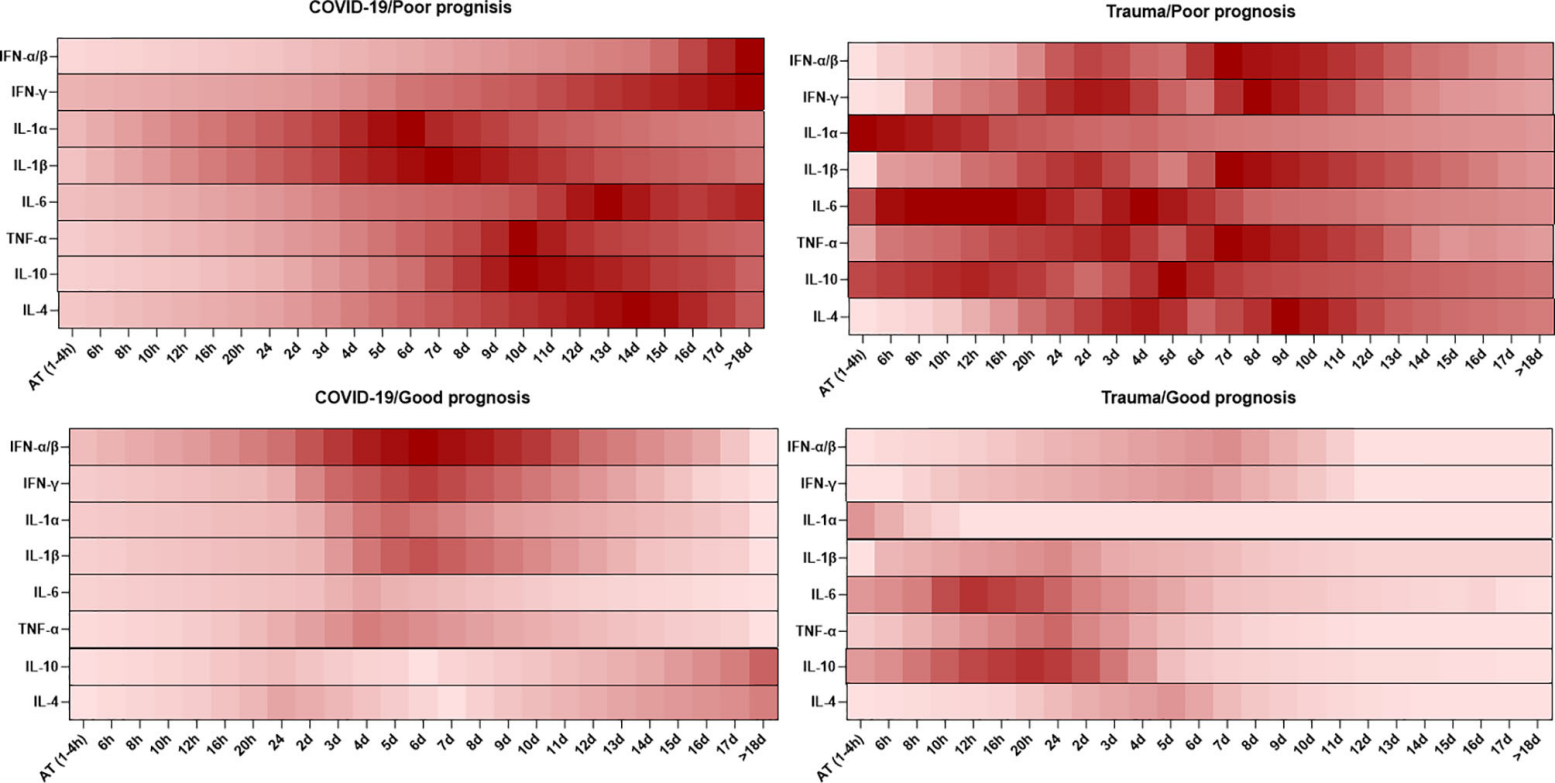
Association between the kinetics of changes in serum levels of cytokines with prognosis in patients with COVID-19 and trauma. The heat map shows the kinetics of changes in serum IFN-α/β (82–86), IFN-γ (86–89), IL-1α (86, 90), IL-1β (86, 90), IL-6 (30, 80, 91), TNF-α (26, 86, 92), IL-10 (26, 80, 91) and IL-4 (86, 92) levels during 18 days after admission in COVID-19 and trauma patients associated with poor and good prognoses. COVID-19 patients with an increased risk of mortality, MOF, severe-to-critical forms of the disease, intensive care unit admission, and/or hospitalization are defined as a poor prognosis for the disease. Conversely, patients with the opposite are defined as having a good prognosis for the COVID-19. AT, admission time; d, day; h, hour; IFN, interferon; IL, interleukin; MOF, multiple organ failure; TNF, tumor necrosis factor.

Cytokines are small molecules released by different types of cells, which display specific functions. Cytokines play vital roles in homeostasis maintenance of the immune system and the pathogenesis of inflammatory diseases (81). The dynamic changes in serum levels of cytokines are associated with prognosis in patients with COVID-19 and trauma, which is represented in **Figure 2**.

#### 3.2.1 IFNs

Type I (especially IFN‐α and IFN‐β) and type II (IFN-γ) IFNs are major members of a big family of cytokines, which are important in protecting against pathogens and tumor cells (93). Type I IFNs, as cytokines of the innate immunity, are produced by virus-infected cells and myeloid dendritic cells. They are at the forefront of defense against viral infections *via* inducing interferon-stimulated genes (ISGs) (94). Whereas, type II IFN is a critical cytokine for both innate and adaptive immunity and plays the major role as an activator of mononuclear cells to stimulate the effector function of cytotoxic T lymphocyte (CTL) and natural killer (NK) cells (95, 96). IFN-α and IFN-β are similar in anti-viral activities, whereas IFN-β appears with greater anti-proliferative and apoptotic effects (97). IFN-β-induced transcriptions are reduced over a longer period compared with IFN-α (97), which indicates the effectiveness of IFN-β in chronic viral infections (98). Although IFNs are the key players in driving anti-viral and anti-proliferative responses, they are also involved in orchestrating the inflammatory condition (99). Kinetic irregularities of IFN-α/β and IFN-γ are associated with poor prognosis in viral and inflammatory diseases (99).

Increased levels of IFN-α 8 to 10 days after the onset of COVID-19 symptoms, and a decrease after day 10, are related to good prognosis. Whereas an opposite pattern appears with a lack of infection control and advanced symptoms (82). IFN-β (both -β1a and -β1b) could facilitate early phase virus clearance (the first 14 days) and improve the survival rate by increasing endothelial barrier activity and anti-inflammatory mediators. It is also likely that IFN-β leads to adverse complications in the late phases (the second 14 days) by promoting inflammation (83–85). Type I IFNs predict positive results in the improvement of SARS-CoV-induced complications in the early phases (100), whereas a high level of IFN-γ at admission that persists in the following days can lead to poor outcomes in COVID-19 patients (87). Only one study has shown IFN-γ serum levels significantly decreased in patients with severe COVID-19 in comparison to cases with moderate symptoms, which is likely due to a decrease in IFN-γ producing lymphocytes (100). Other studies have shown higher serum levels of IFN-γ in patients with severe symptoms (7, 101). Moreover, an increase in serum concentration of this cytokine at days 4 to 6 after the onset of disease symptoms, which decreases over the next few days, is associated with good prognosis (88). It appears a high expression of IFN-γ at admission, which continues for 3 to 4 weeks, could cause negative consequences due to over-activity in the immune system, inducing ACE2 expression (the binding site for SARS-CoV-2) and promoting virus replication (87). Anti-IFN-I autoantibodies have recently been detected in COVID-19 and are associated with poor prognosis of the disease. Bastard et al. have shown at least one type of anti-IFN-I autoantibodies were detectable in 13.7% of patients with life-threatening COVID-19 pneumonia, of which 10.2% of patients had autoantibody against IFN-α (3.6%), IFN-ω (1.3%), or both (5.3%), at the onset of critical disease (102, 103). Anti-IFN-I autoantibodies were higher in males compared to females. Whereas they were found in none of the patients with asymptomatic or mild COVID-19 and were only detectable in 0.3% of healthy individuals aged 20 to 65 years (102). Remarkably, the production of anti-IFN-I autoantibodies is related to the late phase of COVID-19 (104). In the first two weeks post-infection, it is unlikely that the immune tolerance to IFN-I is lost and high titers of autoantibodies are produced (102). This suggests congenital errors of type I IFN production and function. IFN-β therapy may be effective in COVID-19 patients who have anti-IFN-I autoantibodies because auto-IFN-β antibodies are rarely produced.

IFN-α/β and IFN-γ can be prognostic indicators in trauma patients, as they change during the 7 days post- trauma. Levels elevate sharply after severe trauma in two steps; early elevations of IFN-α/β and IFN-γ can be seen 24-72 hours after severe trauma and are correlated to early complications, whereas secondary elevations of IFN-α/β and IFN-γ after 7 days can be an index for opportunistic bacterial infections and sepsis, which are associated with late complications of severe trauma (86). High concentrations of IFN-α/β, IFN-γ, and other pro-inflammatory cytokines in severe trauma, are not only associated with systemic inflammation and tissue destruction in the early phase (the first 14 days), but are also correlated with high levels of anti-inflammatory immune responses in the late phase (the second 14 days). This causes immune deficiency and increases the probability of opportunistic infections (105, 106).

#### 3.2.2 IL-1

IL-1α and -1β are central cytokines of innate immunity, which are produced by hematopoietic, endothelial, and epithelial cells (107). IL-1α increases immune cells migration (108), local and systemic production of inflammatory mediators (109), and lymphocytes proliferation and activation (109). Whereas IL-1β is considered as an effective cytokine on severe complications of inflammatory diseases (25, 110). Even though IL-1β requires processing by inflammasomes for activity, IL-1α can be active in its full-length form without previous processing (25, 111).

Among COVID-19 patients, one study showed no association between IL-1α/β serum levels and disease severity (112). In contrast, other studies have revealed higher levels of IL-1α/β at admission in severe and non-survivor patients compared to mild and survivor cases (7, 101). Serum levels of IL-1β in patients with severe symptoms are approximately 2–100 times above normal levels (113). These conflicting findings may be due to a highly dynamic expression of inflammatory mediator genes in COVID-19 patients. It is likely that different sampling times during the disease course could be a reason for the observed discrepancies among studies. A case-series study in patients with critical-to-severe COVID-19 has shown that most inflammatory genes reach their highest expression levels after the lowest respiratory system function (days 5 post-disease onset) (90). This is in contrast to the role of the CS hypothesis in the pathogenesis of COVID-19. The only expression of IL-1α, IL-1β, IL1 receptor and their signaling pathway molecules had been induced before the lowest respiratory system function (up to 3-5 days after the onset of COVID-19 symptoms) in patients with poor outcomes (90). This shows that the expression of IL-1α, IL-1β, and its signaling pathway molecules increase within 3-5 days post disease onset and might be positively associated with poor prognosis of COVID-19. SARS-CoV-2 induces releasing of IL-1 in macrophages and histamine in mast cells through stimulating toll-like receptor (TLR)2, TLR3, or TLR4 in the early phase of COVID-19 (25, 114). IL-1 affects mast cells and induces the production of IL-6, whereas histamine is involved in the expression of IL-8 and IL-6 in endothelial cells. Besides, histamine in combination with IL-6 stimulates excessive releasing of IL-1, IL-6, and IL-8 from macrophages, which appears as a CS (25, 115). IL-1 also elevates the production of nitric oxide, histamine, metalloproteinases, proteolytic enzymes, and cyclooxygenase (COX)-2 eicosanoid products, such as prostaglandins and thromboxane A2 (TxA2) in mast cells (116), which together with pro-inflammatory cytokines cause septic shock, metabolic dysfunction, thrombi formation, and different tissue damage that can lead to death (25, 116).

DNA microarray analyses of pathological specimens demonstrate that the expression of IL-1α is increased very early after blunt chest trauma (117), but another study has shown a decrease in levels of IL-1α in the plasma of trauma patients at admission (118). These conflicting findings may be due to the rapid fluctuation of IL-1α expression in the early hours after trauma. A very early increase of IL-1α along with elevated levels of IL-1β within 12 to 48 hours post-trauma, is associated with poor prognosis (117). Similar results have been observed in adult acute respiratory distress syndrome (ARDS) and heart and brain ischemia (119–121). Over-expression of IL-1α/β in alveolar macrophages during ARDS (119) and over-production of IL-1α (but no IL-β) in platelets during heart and brain ischemia stimulates the migration of inflammatory cells through inducing the expression of chemokines and adhesion molecules on endothelial cells. These processes could be one of the major mechanisms of type I interferons-mediated organ injuries (120, 121).

#### 3.2.3 IL-6

IL-6 is an acute-phase protein that plays both pro and anti-inflammatory roles in the immunopathology of inflammatory diseases (122, 123). The pro-inflammatory roles of IL-6 run through promoting acute-phase proteins (124), other pro-inflammatory cytokines (125), monocyte/macrophage chemoattractants, and T and B cells proliferation and differentiation (126, 127). Meanwhile, the anti-inflammatory mechanisms of IL-6 are through induction of the anti-inflammatory cytokines and controlling the level of pro-inflammatory cytokines (91, 128, 129).

Several studies have shown that over-production or over-function of IL-6 can be associated with poor prognosis in SARS-CoV-2 infections (45, 130, 131). Hence, serum levels of IL-6 are higher (100–10000 pg/mL) in COVID-19 patients compared to healthy controls. Moreover, COVID-19 patients with more severe symptoms have higher concentrations of IL-6 at admission (30, 132–136). Two independent studies have demonstrated that admission IL-6 serum levels are more likely to be an effective predictor of poor outcomes such as ARDS (optimal cutoff: 80 pg/mL) and death (optimal cutoff: 86 pg/mL) compared to other indicators (134, 137). However, IL-6 levels along with other laboratory indicators, like CRP, prothrombin time (PT), and D-dimer, provide a more accurate assessment of complications in COVID-19 patients (134). Consequently, the combination of a high serum level of IL-6, a high level of D-dimer, and a low level of PT, is associated with DIC-dependent deaths, meanwhile the combination of a high serum level of IL-6, D-dimer, and PT is associated with ARDS-dependent deaths (100, 138). It has been demonstrated that increased levels of IL-6 from 6 hours until ≥18 days post-COVID-19 symptom onset, is associated with poor prognosis (30, 132), whereas its gradual increase within 3-5 days, and its return to normal levels after 15-17 days, is linked to good prognosis (132).

IL-6 may predict post-traumatic complications with high accuracy (122). In homeostatic conditions, serum levels of IL-6 are less than 5 pg/mL. In inflammatory conditions such as trauma, the concentration rapidly rises within hours, reaching the highest levels after 6 to 12 hours (139). Several studies have shown that serum IL-6 concentrations increase in two time periods post-trauma. IL-6 levels in the first 24 hours after trauma are correlated with trauma severity (first hit), whereas its levels during the 48-72 hour period following trauma can be attributed to secondary effects such as infections, surgery, transfusions, and pre-traumatic conditions (91, 140–143).

#### 3.2.4 TNF-α

During the acute phase of inflammation, TNF-α is produced primarily by monocytes/macrophages, but it can also be released by other cells (144). TNF-α plays an essential role in inflammation by inducing other pro-inflammatory cytokines such as IL-1 and IL-6, production of acute-phase proteins, and expression of adhesion molecules (144). The attachment of leukocytes to the endothelium of lymphoid organs and inflammatory sites occurs as a result of the expression of adhesion molecules, which influences the frequency of immune cells in blood circulation and promotes MOF (145). Furthermore, previous research has shown that the serum TNF-α level rises approximately 12 hours after a viral infection and remains elevated for 72 hours (146).

TNF-α levels in the serum of COVID-19 patients increase at the time of admission (3-5 days after infection), which continue until day 3 after admission. Although increased serum TNF-α levels during the first three days after the onset of disease symptoms are associated with disease severity, there are no significant differences in serum TNF-α levels among COVID-19 patients with mild, severe, and critical symptoms. The kinetics of TNF-α after day 3 is different depending on disease progression in patients (132, 147). As a result, higher and lower levels of TNF-α after day 3 are associated with poor and good COVID-19 outcomes, respectively (26, 148).

A positive correlation between serum levels of TNF-α and trauma severity has been indicated in injured patients (149). In non-severe trauma, low amounts of TNF-α are produced, which stimulate tissue repair by inducing fibroblast growth (149, 150). In contrast, excessive increase levels of TNF-α after severe trauma lead to aggravation of inflammatory and MOF (151). High levels of TNF-α and its receptors have been reported in mild trauma 2-4 hours after trauma, peaking at 24-48 hours, and gradually returning to the normal levels after about 5 days (149, 152, 153). However, high serum levels of TNF-α from 2-4 hours until 3 days post-trauma followed by a secondary elevation after day 7, may be associated with poor prognosis of trauma (152, 153).

#### 3.2.5 IL-10

In addition to pro-inflammatory responses, which are one side of the coin in inducing poor outcomes in inflammatory diseases, anti-inflammatory responses followed by these pro-inflammatory responses may also be present. In non-severe inflammatory conditions, there is good balance between these two sides, and inducing low levels of inflammation may promote the repairing process and manage tissue injuries (154). Over-activation of the immune system in severe inflammatory situations can cause an imbalanced immune response and may lead to early complications and MOF in patients. Furthermore, the release of large quantities of anti-inflammatory mediators to control inflammation, causes IS that might induce opportunistic infections and inflammation-dependent late complications (26, 155).

There is reported to be a higher level of IL-10 in COVID-19 patients compared to healthy controls, which is correlated with IL-6 concentrations and disease severity. In this regard, serum IL-10 levels are higher in COVID-19 patients with critical, severe, and moderate symptoms (132). The results have shown serum levels of IL-10 may be a prognostic factor for disease complications (132). Of course, dynamic changes in serum IL-10 levels may predict different outcomes in COVID-19 patients (26). During the first three days after the onset of symptoms, there are no significant differences in serum levels of IL-10 between non-survivor and survivors groups of COVID-19 patients. The significant differences start during days 4 to 7 post-symptom onset and continue until days 8 to 13. An increase of serum IL-10 levels within 4 to 19 days after initial symptoms of disease, and a decrease in the days following, is associated with poor outcomes, whereas an opposite pattern is observed in patients with good outcomes (26). Thus, serum level of IL-10 4 to 13 days following COVID-19 might be considered as another prognostic indicator.

A significant increase in serum IL-10 levels can be seen within 24 hours post-trauma (156). An early but slow increase of serum IL-10 is related to the low production of pro-inflammatory mediators after non-severe trauma and can be a regulating and repairing factor in the early phase of inflammation. Although IL-10 has the ability to modulate inflammatory responses, it has been linked to poor prognosis in severe injuries (156). Very high levels of IL-10 within 2-24 hours after trauma are linked to high levels of pro-inflammatory cytokines, which is caused by the intensity of the injury (first hit) and leads to immunodeficiency (91, 129, 139). This phenomenon increases susceptibility to opportunistic infections and sepsis (second hits), resulting in a higher secondary pro-inflammatory response and IL-10 rise after 72 hours (91, 157). Secondary elevation of IL-10 can be a poor prognosis of common bacterial infection, sepsis, and MOF in trauma patients (91).

#### 3.2.6 IL-4

IL-4 is produced mainly by activated type 2 helper T (Th2) cells and regulates cell apoptosis and proliferation, Th1 responses, Th2 differentiation, isotype switching to immunoglobulin E (IgE), and skewing of macrophages toward type 2 macrophages (M2MQ) (158). Respiratory infections have been shown to increase serum levels of IL-4 as an anti-inflammatory cytokine (159).

Serum levels of IL-4 are observed to be much higher among COVID-19 patients compared to healthy controls (132). In particular, higher levels of IL-4 have been reported in COVID-19 patients with more severe respiratory symptoms (7, 46). Despite this observation, a multivariable comparison has shown no significant differences between patient groups with mild, severe, and critical symptoms (132). The kinetics of IL-4 are different in COVID-19 patients with different illness severity. A decrease in serum IL-4 levels during the first week after disease onset and a gentle increase during the second week is associated with good prognosis of COVID-19 (92). Whereas, the contrary trend is associated with poor COVID-19 outcomes (92).

Serum levels of IL-4 are significantly higher in trauma patients compared to healthy controls and positively correlated with trauma severity (160). However, previous studies have reported controversial results from IL-4 levels in patients with and without post-traumatic complications (161, 162). A study on trauma patients with high serum levels of IL-6 showed that the expression of IL-4 in trauma patients with post-traumatic complications is lower than in cases without complications (163). Conflicting findings may be due to different sampling times during the disease. It seems that the correlation between IL-4 and IL-6 in the incidence of post-traumatic disorder is similar to the correlation between IL-10 and IL-6 (91, 129). Accordingly, the synchronic increase and decrease of both IL-6 and IL-4 may be related to the control of inflammatory responses in early and late phases after trauma, respectively (160). Whereas an imbalance between IL-6 or IL-4 may lead to early or late complications (160). There is limited data on the kinetics of post-traumatic IL-4; if accurately determined, it may lead to a prognostic factor for predicting post-traumatic events.

### 3.3 Immune Cells

The incidence of leukocytosis, neutrophilia, lymphopenia, and thrombocytopenia in inflammatory conditions such as infectious disease (7, 130), and trauma (164, 165), might be attributed to different mechanisms. This includes: migration of mononuclear cells to inflammatory tissue, release of neutrophils from the bone marrow, inhibition of lymphocyte proliferation by acidosis, inducing Fas/Fas ligand (FasL)-dependent apoptosis of lymphocytes under effect the high level of IL-6, inhibition of lymphocyte recirculation due to the strong attachment of lymphocytes to the lymphoid organs endothelial, and lymphoid organ atrophies that may be related to the lymphocytes exhaustion (4).

**Figure 3:**
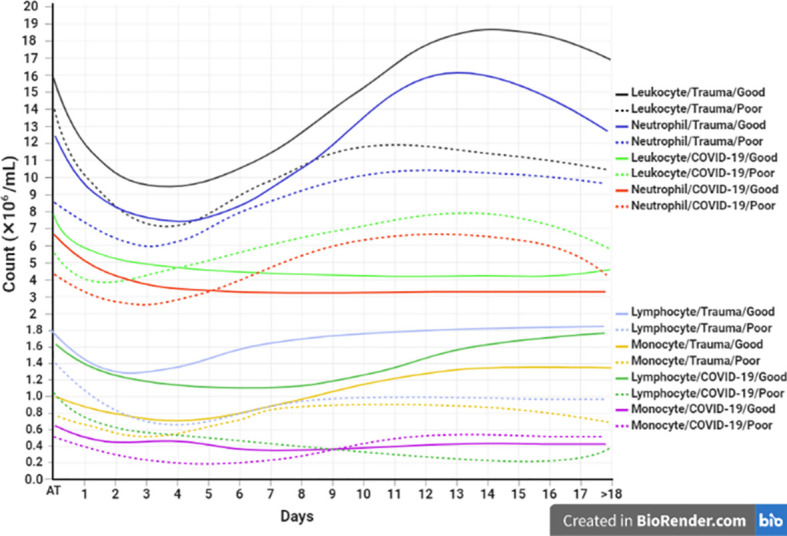
Association between the kinetics of changes in blood cell count with prognosis in patients with COVID-19 and trauma. The timeline plot represents the kinetics of changes in leukocyte (30, 166, 167), neutrophil (30, 168), lymphocyte (30, 45, 167, 169, 170), and monocyte (30, 166, 171) counts during 18 days after admission in COVID-19 and trauma patients associated with good (solid lines) and poor (dashed lines) prognoses. COVID-19 patients with an increased risk of mortality, MOF, severe-to-critical forms of the disease, intensive care unit admission, and/or hospitalization are defined as a poor prognosis for the disease. Conversely, patients with the opposite are defined as having a good prognosis for the COVID-19. MOF, multiple organ failure.

Laboratory indicators such as increased neutrophil/lymphocyte ratio (NLR) and platelet/lymphocyte ratios (PLR), as well as decreased lymphocyte/WBCs ratio (LWR), could be a useful predictor in the prognosis of COVID-19 and trauma (132, 138, 166). Besides, investigation of similarities and differences in each immune cell subtype between patients with COVID-19 and trauma might be very interesting for the prediction of disease complications. The dynamic changes of blood circulating cells are associated with prognosis in patients with COVID-19 and trauma as represented in **Figure 3**.

#### 3.3.1 Innate Immune Cells

##### 3.3.1.1 Neutrophil

Neutrophils contribute to the pathogenesis of inflammatory diseases by ROS production, neutrophil extracellular traps (NETs) formation, inducing RBCs dysfunction, and promoting thrombosis (172). Infiltration of neutrophils into alveolar spaces causes transient neutropenia within days 1-5 post-disease onset in COVID-19 patients with severe symptoms. This process is reversed approximately after 5 days of the beginning of COVID-19 symptoms, which is due to the release of neutrophils from the bone marrow. Neutrophilia can become evident in peripheral circulation within days 9 to 11, and continues until days 15 to 17 after symptom onset (30, 173). A significant correlation between the increased number of neutrophil cells and severe infection from COVID-19 has been documented (174). Similarly, increased NLR and NETs have been reported in the peripheral blood of patients with critical symptoms (175, 176). It has been indicated that NLR<3 correlates with better clinical outcomes, whereas NLR≥4 is a predictor of ICU admission and disease severity (177). It seems that neutrophilia, especially during 9 to 17 days after initial symptoms of disease, is a poor prognostic indicator and could be considered an independent risk factor for early screening of COVID-19 patients with severe symptoms.

Studies have shown neutrophil count is increased within 4 to 6 hours following trauma and contributes to tissue repairing through different mechanisms, including phagocytosis of cell debris, and releasing NETs, serine proteases, cytokines, and chemokines (178). Since neutrophils are the first responders to tissue injuries, the expression levels of CD11b and CXCR2 are considered important indicators of trauma injury prognosis (179, 180). High serum levels of IL-8 and over-expression of its receptor (CXCR2) on the neutrophils in patients with severe trauma have been reported, which correlate with neutrophil hyperactivity and poor prognosis (178, 181). Moreover, high levels of NLR (>5.27) over 24 to 48 hours post-trauma are significantly correlated with early MOF (182) and mortality (183). Hence, the level of CD11b and CXCR2, as well as NLR at the time of admission, may be valuable predictors to identify the inflammatory state and risk of mortality in trauma patients.

##### 3.3.1.2 Monocytes

Monocytes are key cells of the innate immunity in the initiation, maintenance, and resolution of inflammation through three major functions include phagocytosis, antigen presentation, and immunomodulation (184). In COVID-19, monocytes are the major players in inducing the inflammatory response and CS in patients with severe symptoms (185). Phenotypic changes in peripheral blood monocytes of SARS-CoV-2 infected patients are correlated with different prognoses (186). In this regard, increased frequencies of CD14+ CD16+ monocytes in the peripheral blood of patients with severe COVID-19 have been observed (187, 188). In the first 5 days after symptom onset, total monocyte counts decrease more sharply in patients with severe symptoms than mild cases, regardless of phenotype. This trend is reversed approximately from days 5 to 9 onwards, which may be the same as CD14+ CD16+ monocytes (30). The significant difference between patients with mild and severe symptoms is observed on days 3 to 5 after the onset of disease symptoms (30), which can be used as an indicator to predict COVID−19 prognosis.

Likewise, monocyte roles in trauma injuries are more widely recognized (189). Previous studies have shown that monocyte counts immediately increase in the acute phase of stroke (190, 191). Furthermore, it was recently found that CD14+ monocyte counts increased after surgical trauma and reach a peak in the first week (192). Zhiqi et al. reported a significant correlation between monocyte counts and 6 month outcomes in patients with moderate-to-severe traumatic brain injuries (193). Others have shown that monocyte dysfunction, decreased TNF-α secretion, and increased anti-inflammatory cytokines production are correlated with higher mortality in patients with severe trauma (194–196). It seems that a gentle decrease in monocyte counts during 4 days after trauma and a mild increase in their counts during days 5-14 correlates with better prognosis. A sharp decrease of monocyte counts during the period 3 days following trauma, which increases severely during days 5 to 7 and decreases after day 8, could indicate a poor prognosis.

#### 3.3.2 Adaptive Immune Cells

Lymphocytes including T, B, and NK cells play a pivotal role in the humoral and cellular immune response against viral infections (197). Previously, changes in the peripheral blood lymphocyte subsets have been observed in several respiratory infections caused by RNA viruses (198, 199). A reduction of the lymphocyte count, especially total T, CD4+ T, CD8+ T and NK cells along with neutrophilia and thrombocytopenia are typical phenomena following SARS-CoV-2 infection (45, 200, 201). In mild COVID-19 the decreased lymphocyte count is within the normal range, whereas it appears as lymphopenia in severe COVID-19 (165, 202, 203). In patients with severe COVID-19, lymphopenia appears within 2 days after initial symptoms and returns to normal ranges after day 18, whereas it persists for a longer time in non-survivor patients (204). Several studies have reported a state of lymphopenia in CD4+ T, CD8+ T, B and NK cells in COVID-19 patients (205, 206), however others have shown higher reductions of CD8+ T than that CD4+ T cells (207, 208). Therefore, counting the number of lymphocytes within 21 days after symptoms onset could be a prognostic indicator for future complications of COVID-19. The trend of changes in lymphocyte count in trauma patients differs from that in patients with COVID-19 (**Figure 3**).

It has been shown that lymphocyte counts decrease immediately after trauma in patients compared to the control group (167, 209), although significant decreases in the number of lymphocytes between MOF and non-MOF groups appear after day 2 (169). Accordingly, lymphopenia is detectable during days 2 to 7 after trauma in the MOF group. Whereas in non-MOF patients, lymphocytes decrease to the lowest counts of the normal ranges on the second day after trauma, and then gradually increase (169). The persistence of lymphopenia following trauma is correlated with severity and is associated with poorer prognosis.

##### 3.3.2.1 CD4+ T Cells

CD4+ T cells are key orchestrators of adaptive immune responses. In the early phase of COVID-19, a dramatic decrease of CD4+ T cells is observed, which correlates with COVID-19 severity (173). Sharp depletion of CD4+ T cell count 1 to 3 days post-symptom onset, which is followed by a slight decrease until days 16 to 20, is associated with poor prognosis (<300/μL) (158). The persistence of this reduction after days 16 to 20 has a strong correlation with mortality, but its elevation is accompanied by the recovery of patients from COVID-19 (158). Furthermore, the expression of activation and/or exhaustion markers by CD4+ T cells, have been observed in patients with severe COVID-19 symptoms (210). In this regard, SARS-CoV-2-specific HLA-DR+ Ki-67+ PD-1+ CD4+ T cells are observed in COVID-19 patients (211).

In trauma patients, changes in CD4+ T cell counts and total lymphocyte counts are similar to SARS-CoV-2 infection. Limited findings have revealed a significant decrease in CD4+ T cell count 3 days after injury in trauma patients (167). Another study has shown CD4+ T cell loss is associated with adverse outcomes after septic shock (212). However, further evaluation is required to determine exact kinetic changes of CD4+ T cells count post-trauma.

**Figure 4:**
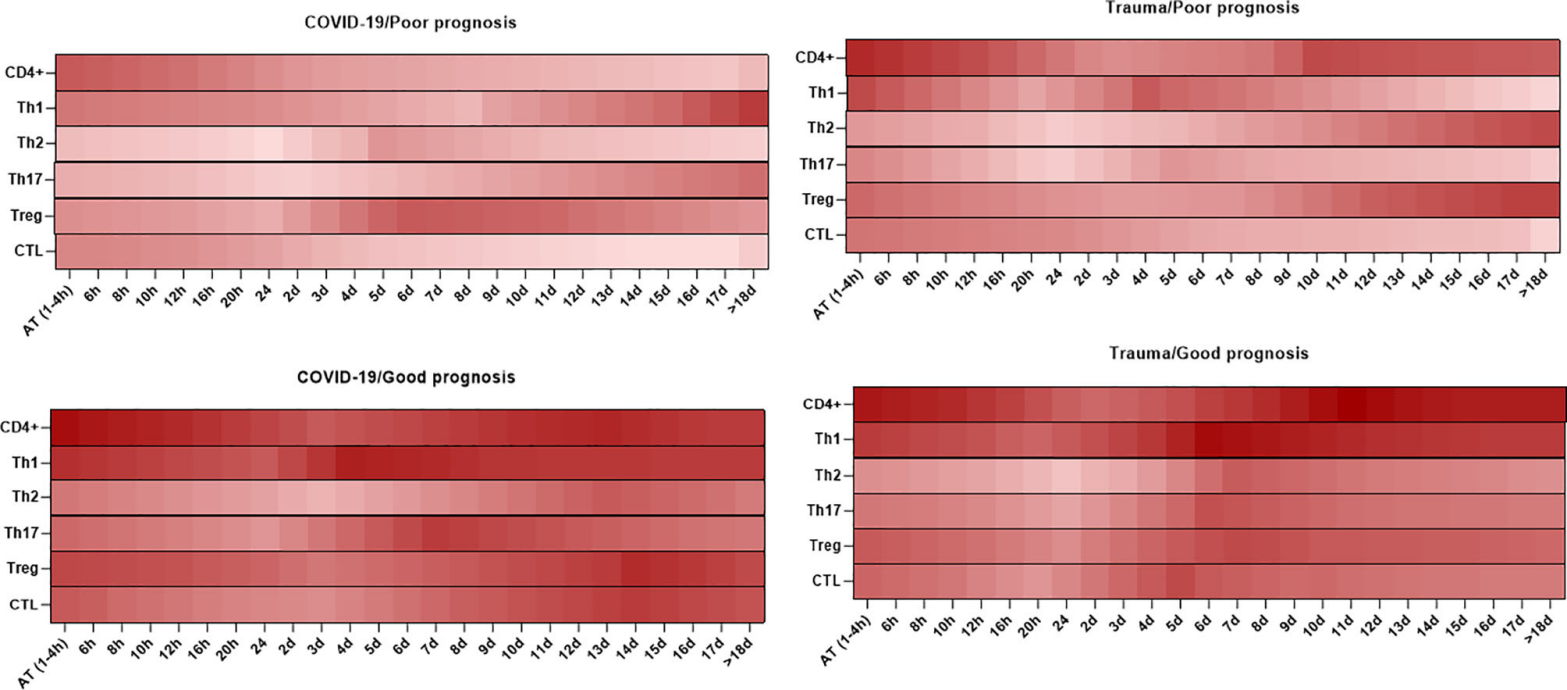
Association between the kinetics of changes in circulating CD4+ and CD8+ T cell subsets with prognosis in patients with COVID-19 and trauma. The heat map shows the kinetics of changes in CD4+ T cells (167, 170, 211, 213), Th1 (214, 215), Th2 (214, 215), Th17 (214, 215), Treg (214, 215), and CTL (30, 170, 213, 216–218) counts during 18 days after admission in COVID-19 and trauma patients associated with poor and good prognoses. COVID-19 patients with an increased risk of mortality, MOF, severe-to-critical forms of the disease, intensive care unit admission, and/or hospitalization are defined as a poor prognosis for the disease. Conversely, patients with the opposite are defined as having a good prognosis for the COVID-19. AT, admission time; CTL, cytotoxic T cell; d, day; h, hour; Th, helper T cell; MOF, multiple organ failure; Treg, regulatory T cell.

As shown in **Figure 4**, there are different kinetics of CD4+ T cell subpopulations, including Th1, Th2, Th17, and Treg cells in peripheral blood of patients with SARS-CoV-2 infection and trauma injuries. These can be used to predict the prognosis of disease as described below (219, 220).

###### 3.3.2.1.1 Th1 Cells

Th1 cells represent an appropriate immune response against viral invasion by releasing pro-inflammatory cytokines such as IL-2, TNF-α, and IFN-γ (221). In the early phase of COVID-19 infection, short-lived, highly functional, and terminally differentiated effector Th1 cells eliminate infected target cells (222). In addition, SARS-CoV-2 spike protein-specific CD4+ T cells with prominent properties of Th1 cytokines profile have been identified in the early phase of COVID-19 (223, 224). Studies have shown that an increase in IFN-γ producing Th1 cells within the first week after COVID-19 symptoms onset is correlated with mild symptoms of COVID-19 (225). Whereas a decrease in IFN-γ producing Th1 cells during the first week and an increase in polyclonal granulocyte-macrophage colony-stimulating factor (GM-CSF) IL-6 producing Th1 cells within the second week of disease are associated with severe symptoms (225). It has also been indicated that increased IFN-γ producing Th1 cells in the late phase of COVID-19 is associated with disease pathogenesis and poor prognosis (226). Recent documents confirm a lower frequency of the cellular component Th1 in the early phase of the severe COVID-19 (135, 227). The Th1 deficiency in the early phase negatively affects the number and function of active CTLs against SARS-CoV-2 at various levels (228). Furthermore, Th2/Th1 imbalance in the early phase and subsequent Th1 exhaustion in the late phase are associated with progression of SARS-CoV-2 infection and poorer prognosis in severe COVID-19 individuals (229, 230). It has been shown that increased expansion of peripheral neutrophils in severe COVID-19 cases potentially suppresses Th1 cells differentiation and triggers Th17 cells polarization in severe patients (231).

Although more research is needed to more accurately determine the kinetics of Th1 cells after trauma, accumulating studies have indicated that IFN-γ producing Th1 responses are significantly reduced after severe trauma (232–235). In contrast, a study in severe thoracic trauma patients has suggested that the frequency of Th1 cells is significantly higher the first week after trauma when compared with healthy individuals. The number of Th1 cells gradually decrease in the following weeks (214). Once again it appears that the differences in study results are due to sampling time differences throughout the disease course. Severe trauma is associated with an increase in Th1 cells in the first week following trauma that may predispose patients to poor outcomes early on. Conversely, a decrease in T cells in the following weeks is associated with immunodeficiency and sepsis, which is followed by poor outcomes observed later in the trauma period (214). However, more studies are required to accurately determine the kinetics of Th1 cells post-trauma.

###### 3.3.2.1.2 Th2 Cells

Extracellular pathogens can trigger Th2 cells immune response (236). Whereas the frequency of CD4+ T cells is significantly lower in patients with COVID-19 compared to healthy controls (158), a recent study identified some functional signals of Th2 cells, such as the degranulation of basophils and eosinophils in hospitalized patients (237). In this scenario, Th2 cells produce cytokines such as IL-4, -5, -10, and -13, which are significantly correlated with disease severity and mortality in COVID-19 patients (238, 239). An increased function and decreased count of Th2 cells could be indicators for COVID-19 outcomes. However, there is little data associating kinetics of frequency and function for Th2 cells and possible future complications in COVID-19 patients.

In the case of trauma, depending on injury severity, circulating effector T lymphocytes change from a pro-inflammatory Th1 phenotype in the early phase to an anti-inflammatory Th2 phenotype in the late phase (240). Data have shown that trauma-associated injuries promote immune response of Th2 cells (214, 241). Kinetically, there is no significant difference in the frequency of Th2 cells in patients with trauma compared to healthy individuals at admission. Conversely, Th2 cells gradually increase in the days following trauma, and significant differences can be observed 2 weeks after admission (214). Hence, an increase in Th2 cells within 2 to 3 weeks of trauma could be a poor prognosis for complications. High levels of anti-inflammatory responses induced by Th2 cells result in immune deficiency, which predisposes individuals to opportunistic infections (9, 242).

###### 3.3.2.1.3 Th17 Cells

In the context of SARS-COV-2 infection, dendritic and endothelial cells by TGF-β, IL-1β, IL-6, and IL-18 secretion and neutrophils *via* nitric oxide synthases induce a Th17 response (231). Although some studies have shown a decrease in the frequency of circulating CD4+ T cells due to localization in the lung and other tissues, other studies have reported an increased number of CCR6+ CD4+ T cells in patients with severe COVID-19 (104, 243, 244). GM-CSF+ IL-6+ CCR6+ Th17 cells have been detected in the blood of COVID-19 patients (225). However, the Th17 response does not effectively control SARS-COV-2 infection, and induces recruitment of neutrophils, thus reinforcing CS and causing more tissue damage (215, 245). Moreover, new findings confirm that high activation of Th17 cells and high signaling of IL-17 are significantly associated with severe COVID-19 (231, 246). A recent study showed exhausted T cell profiles are associated with increased Th17 responses in COVID-19 patients with pneumonia (247). It has been reported that Th17 cells increase during the period 2 to 3 weeks post-symptom onset can predict a poor prognosis for COVID-19 complications. Therefore, this suggests that Th17 cells do not effectively control intracellular microorganisms, and consequently lead to pneumonia and edema by decreasing Treg function, promoting neutrophil migration, and inducing eosinophilic responses (248).

Th17 cells modulate immune response after trauma. Accordingly, high frequencies of Th17 cells have been reported in the peripheral blood of patients with severe trauma on the first week following admission (214, 249). Studies on trauma patients admitted to the ICU ward have identified increased Th17 cells and serum IL-17 levels during the first week after admission, which are correlated with development of early poor outcomes (250, 251). However, the frequencies of Th17 cells are reduced subsequently 2 to 3 weeks after admission in the group with severe trauma, which may potentially lead to complications later (214). So, assessing the frequency and activity of Th17 cells during the first and following weeks after trauma may be useful in predicting early and late complications, respectively.

###### 3.3.2.1.4 Treg Cells

Treg cells are divided into two subtypes, including natural and inducible regulatory T (nTreg and iTreg, respectively) cells, which play an important role in immune tolerance as well as autoimmune and inflammatory disease prevention (252). Both Treg subsets stimulate tissue repair and immune response homeostasis during acute infections (253). Several studies have demonstrated that the frequency of Treg cells increases in patients with mild COVID-19 and recovered individuals (253, 254). Opposite results have been reported in patients with severe COVID-19 (138, 255–257). It seems an increase in the frequency of Treg cells in the early phase and a decrease in these cells in the late phases of COVID-19 are associated with poor outcomes. The former by inhibiting antiviral response and the latter by contributing to excessive pro-inflammatory responses. In this context, a study found that a decrease in Treg cells and an increase in Th17 cells are associated with the uncontrolled release of pro-inflammatory cytokines in COVID-19 patients (258). In combination with TGF-β1, a high level of IL-6 in the sera of patients with severe COVID-19, induces the differentiation of Th17 and inhibits iTreg (TGF-β1-induced Treg) generation (259, 260). Moreover, gene expression analyses in the CD4+ T cells from COVID-19 patients revealed a decrease in IL-2 transcripts in severe COVID-19 cases compared to mild cases, which could be another reason for decreasing Treg cells (261). Little is known about the kinetics of changes in the number of T cells during COVID-19 disease, which is an important area for further research.

Serve et al. have shown that the frequency of both CD4+ CD25+ Foxp3+ Treg and CD4+ CD25+ CD127- cells in trauma patients are lower compared to healthy controls (167). Kinetically, CD4+ CD25+ FoxP3+ Treg cells decrease 4 hours after trauma (262) and continue to decline for up to 72 hours without significant improvement (167). On other hand, there were no significant changes in the frequency of CD4+ CD25+ Treg cells immediately after trauma, but there was a large rise on day 7 after trauma, according to MacConmara et al. (263). Zhang et al. have reported an increase in the frequency of CD4+ CD25+ CD127low/− Treg cells during 3 weeks after severe thoracic trauma (214). Differences in the phenotype intended to identify Treg cells in different studies, phenotypic variability of Treg cells in the sites of trauma, and the stage of the disease at which sampling is performed, are the most important factors influencing the results. It has been observed that the decrease and increase of Treg cells in the early and late phases of the disease, respectively, are associated with poor prognosis of trauma. Thus, similar to COVID-19, Treg cells may be important in the pathogenesis of trauma complications in a competitive pattern with Th17 cells (262).

##### 3.3.2.2 CD8+ T Cell

CD8+ T cells exert their biological activity by inducing apoptosis in adjacent infected cells after releasing cytotoxic granules (255) and secreting cytokines (264). The density of granules and their content such as perforin and granzymes in CD8+ T cells might demonstrate the overall status of cellular immunity (265). Studies have shown the CD8+ T cell counts decrease in all COVID-19 patients with mild, moderate, severe, and critical symptoms compared to healthy controls. CD8+ T cells are strongly reduced in patients with a more severe form of COVID-19 (100, 266–268). In patients with severe COVID-19, the kinetics of CD8+ T cells demonstrate that the lowest count of CD8+ T cells occurs within 3 to 5 days after the onset of disease, and this tendency persists until day 18 (30). The frequency of CD8+ T cells in the non-survivor group decreases until death, but it increases after days 18 to 20 among individuals who improve (158). Hence, determining the cut-off point for T cells, especially CD4+ and CD8+ T cells after the development of COVID-19 symptoms, can be a valuable prognostic indicator and may predict disease progression (158). However, in individuals with severe COVID-19, the frequencies of CD8+ T cells that express activation markers such as HLA-DR and exhaustion markers including PD-1, Tim-3, and NKG2A are higher than in mild cases (16, 225, 255). Thus, activated CD8+ T cells in patients with critical and severe COVID-19 have reduced degranulation and secretion of granzyme B (GrZB) and perforin as compared to healthy donors (225, 243). These findings suggest that higher levels of over-active CD8+ T cells may be harmful in the later stages of the illness due to their excessive pro-inflammatory cytokine production (100). In contrast, SARS-CoV-2-specific HLA-I multimer+ CD8+ T cells from severe COVID-19 patients express activation markers (CD38 and HLA-DR), inhibitory receptors (PD-1, TIM-3, and LAG-3), cytotoxic proteins (GrZB and perforin), and Ki-67, representing that these cells are activated and proliferate with a high cytotoxic capacity. It suggests that PD-1 expression in SARS-CoV-2-specific CD8+ T cells from severe COVID-19 patients is transient and is not associated with decreased cytotoxic dysfunction (269). Similar results observed that the expression of TIM-3 and LAG-3 exhaustion-associated genes was higher in SARS-CoV-2-specific CD8+ T cells from COVID-19 patients (270). Furthermore, another analysis showed that a considerable fraction of the PD-1 expressing SARS-CoV-2-specific multimer+ CD8+ T cells produced IFN-γ, suggesting that this proportion is not exhausted in patients with COVID-19 (271). Overall, these findings reinforce the effects of CD8+ T cells and their activation and exhaustion markers on the severity of COVID-19.

Disorders involving CD8+ T cells can lead to maladaptive immune responses that can cause complications and mortality after severe trauma. Previous studies have found that CD8+ T cell numbers and perforin expression in their granules are lower during the first 24 hours after severe trauma among people experiencing poorer outcomes (MOF and mortality groups), and the trend continues up to day 7 (216–218). In addition, the frequencies of GrB+ CD8+ T cells in the poorer outcome group are decreased during days 3 to 7 post-trauma (216). A secondary decrease is observed in week 4 after severe trauma, leading to development of opportunistic infections (217). It suggests that immune responses occur rapidly after trauma within a few days. Excessive and frequent activation of T cells (both CD4+ and CD8+ T cells) can cause apoptosis (272) and exhaustion (273), resulting in a decrease in their number and function, which are associated with the occurrence of post-trauma opportunistic infections. As a result, CD8+ T cells counts and expression levels of their perforin and GrB within the first 7 days after trauma could be considered a valuable prognostic indicator for predicting trauma outcomes.

## 4 Challenges and Future Directions

Many immune mediators have been identified as prognostic indicators for COVID-19. However, their presence in isolation does not yield accurate or rapid prediction for COVID-19 outcomes in the context of other inflammatory diseases. Therefore, universal profiling is required. In addition, due to the dynamics of immune mediators in the presence of inflammation, several investigations have produced contradictory results. Hence, further evaluation to determine the best sampling time and reference ranges may be necessary to achieve more accurate results. The phenotypic diversity of immune cells under different inflammatory conditions is another challenge that affects the results and highlights the need to define a standard phenotypic pattern to detect immune cells. Other problems include confounding variables such as age, gender, and comorbidities, which can alter immune mediators as targets for predicting disease prognosis and should be considered. Another problem is the direct effects of genetics on immune system responses in inflammatory conditions. Thus, determining the genetic properties associated with anti-SARS-CoV-2 immune responses is extremely helpful in identifying the pathogenesis of immune factors. Furthermore predicting disease prognosis will be less useful without an initial estimation of viral infectious dose or replication power, which should be considered.

## 5 Concluding Remarks

Immune system mediators, both molecular and cellular, might be considered as prognostic candidates for COVID-19 outcomes. Although prognostic properties of these mediators for COVID-19 complications are impaired under the effect of the simultaneous presence of other inflammatory diseases such as trauma, estimating the most appropriate evaluation time of their kinetics to determine the differences between COVID-19 and trauma might be useful in accurately predicting COVID-19 outcomes in the context of trauma. Hence, molecular and cellular mediators analyses associated with COVID-19 prognosis in the context of trauma can be performed at the time of admission. Since patients with COVID-19 usually are referred to the hospital 3 to 5 days after SARS-CoV-2 infection, the amounts of many mediators have changed significantly. Whereas for individuals with traumatic injuries this time is 1 to 4 hours after trauma. Thus, evaluating a large number of mediators that begin to change at least after 4 to 6 hours can be a useful candidate for predicting COVID-19 prognosis in the setting of trauma. PCT, ferritin, TNF-α, and IL-1α are molecular mediators of the immune system where concentrations are altered immediately post-trauma and therefore their evaluation at admission is not reliable to predict COVID-19 outcomes in the context of trauma. Other immune systems mediators are therefore recommended.

In mild COVID-19 and non-severe trauma, after an insignificant local inflammation in the early phase, the increase in inflammatory mediators is immediately controlled by the synthesis of anti-inflammatory mediators and followed by tissue repair. In severe trauma (“first hit”), high local inflammation in the early phase is associated with early SIRS, MOF, and mortality, as is a correlation with CARS, resulting in immune deficiency. The former by over-activation of immune responses and waste of the immune system energy, and the latter through over-suppression of the immune system due to PICS, which increases the risk of opportunistic infections (“second hit”). Infections lead to late poor outcomes by promoting more severe forms of SIRS, CARS, and PICS. Many suboptimal outcomes in both the early and late phase of severe COVID-19 are caused by hyper-inflammation, also known as the CS.

## Author Contributions 

HF, MB, and SS conceptualized and designed the study. HF, AT, NE, MN, and BG wrote the first draft of the manuscript. NE, SN, KC-C, A-AK, and SS critically revised the manuscript. All authors reviewed and approved the final version of the manuscript.

## Funding

The present study was funded by Shahid Beheshti University of Medical Sciences, Tehran, Iran (grant number: 30102).

## Conflict of Interest

The authors declare that the research was conducted in the absence of any commercial or financial relationships that could be construed as a potential conflict of interest.

## Publisher’s Note

All claims expressed in this article are solely those of the authors and do not necessarily represent those of their affiliated organizations, or those of the publisher, the editors and the reviewers. Any product that may be evaluated in this article, or claim that may be made by its manufacturer, is not guaranteed or endorsed by the publisher.

## Glossary

**Table:**

AA	amyloid A
ACE	angiotensin-converting enzyme
ARDS	acute respiratory distress syndrome
CARS	compensatory anti-inflammatory response syndrome
CCR	chemokine (C-C motif) ligand receptor
COVID-19	coronavirus disease-2019
CRP	c-reactive protein
CS	cytokine storm
CT	computerized tomography
CTL	cytotoxic T lymphocyte
CXCR	chemokine (C-X-C motif) ligand receptor
COX	cyclooxygenase
DAMP	damage-associated molecular pattern
DIC	disseminated intravascular coagulation
FasL	fas ligand
GM-CSF	granulocyte-macrophage colony-stimulating factor
GrZB	granzyme B
HDL	high-density lipoproteins
HLA	human leukocyte antigen
ICU	intensive care units
IFN	interferon
IL	interleukin
IgE	immunoglobulin E
IS	immunosuppression
ISG	induction of IFN-stimulated gene
ISS	injury severity score
iTreg	inducible regulatory T
LDL	low-density lipoproteins
LWR	lymphocyte-to-WBCs ratio
MOF	multiple organ failure
M2MQ	type 2 macrophages
NETs	neutrophil extracellular traps
NK	natural killer
NLR	neutrophil-to-lymphocyte ratio
nTreg	inducible regulatory T
PCT	procalcitonin
PD-1	programmed cell death protein-1
PICS	persistent inflammation, immunosuppression, and catabolism syndrome
PLR	platelet-to-lymphocyte ratios
PT	prothrombin time
ROS	reactive oxygen species
RT-qPCR	real time-quantitative polymerase chain reaction
SAA	serum amyloid A
SARS-CoV	severe acute respiratory syndrome coronavirus
SIRS	systemic inflammatory response syndrome
TBI	traumatic brain injury
TGF	transforming growth factor
Th	helper T cell
Tim-3	T-cell immunoglobulin mucin 3
TLR	toll-like receptor
TNF-α	tumor necrosis factor
Treg	regulatory T cells
TxA2	thromboxane A2
WBC	white blood cell
