# Supplementary material for: Potential Immune Indicators for Predicting the Prognosis of COVID-19 and Trauma: Similarities and Disparities
Source: Front Immunol. 2022 Jan 20;12:785946. doi: 10.3389/fimmu.2021.785946 (PMC8815083; doi:10.3389/fimmu.2021.785946)
Supplement: Supplementary file 1 [file DataSheet_1.docx]

**References for Figures**

**CRP/COVID-19**

<https://doi.org/10.1007/s00430-020-00693-z>. (1)

<https://doi.org/10.1002/jmv.25871>.

<https://doi.org/10.2147/RMHP.S268238>. (2)

<https://doi.org/10.1080/22221751.2020.1770129>. (3)

**CRP/Trauma**

<https://doi.org/10.3109/17453679809117625>. (4)

**PCT/COVID-19**

<https://doi.org/10.1007/s00430-020-00693-z>. (1)

<https://doi.org/10.2147/RMHP.S268238>. (2)

**PCT/Trauma**

<https://doi.org/10.3892/etm.2019.7492>. (5)

<https://doi.org/10.1186/cc3910>. (6)

<https://doi.org/10.1007/s001340051006>. (7)

<https://doi.org/10.1097/00003246-200004000-00007>. (8)

<https://doi.org/10.1097/TA.0b013e31825ff5b7>. (9)

**Ferritin/COVID-19**

<https://doi.org/10.2147/RMHP.S268238>. (2)

**Ferritin/Trauma**

<https://doi.org/10.1038/srep21970>. (10)

**SSA/COVID-19**

<https://doi.org/10.3892/etm.2020.9114>. (11)

**SSA/Trauma**

<https://doi.org/10.1177/0885066619837913>. (12)

<https://doi.org/10.1155/2019/5967816>. (13)

<https://doi.org/10.1089/neu.2019.6570>. (14)

**IFN-αβ/COVID-19**

<https://doi.org/10.1016/j.jaci.2020.04.029>. (15)

<https://doi.org/10.1128/AAC.01061-20>. (16)

<https://doi.org/10.1016/S0140-6736(20)31101-6>. (17)

<https://doi.org/10.1016/j.antiviral.2020.104791>. (18)

**IFN-αβ/Trauma**

### <https://doi.org/10.1097/SLA.0000000000001001>. (19)

**IFN-γ/COVID-19**

<https://doi.org/10.1016/j.ebiom.2020.102763>. (20)

<https://doi.org/10.15252/emmm.202013191>. (21)

**IFN-γ/Trauma**

<https://doi.org/10.1523/JNEUROSCI.17-10-03664.1997>. (22)

### <https://doi.org/10.1097/SLA.0000000000001001>. (19)

**IL-1α/COVID-19**

<https://doi.org/10.1016/j.chom.2020.03.021>. (23)

**IL-1α/Trauma**

### <https://doi.org/10.1097/SLA.0000000000001001>. (19)

**IL-1β/COVID-19**

<https://doi.org/10.1016/j.chom.2020.03.021>. (23)

**IL-1β/Trauma**

### <https://doi.org/10.1097/SLA.0000000000001001>. (19)

**IL-6/COVID-19**

<https://doi.org/10.1080/22221751.2020.1770129>. (3)

<https://doi.org/10.1007/s00430-020-00693-z>. (1)

**IL-6/Trauma**

<https://doi.org/10.1155/2015/747036>. (24)

**TNF-α/COVID-19**

### <https://doi.org/10.1038/s41392-020-0211-1>. (25)

### <https://doi.org/10.21037/apm-20-2134>. (26)

**TNF-α/Trauma**

### <https://doi.org/10.1097/SLA.0000000000001001>. (19)

**IL-10/COVID-19**

### <https://doi.org/10.21037/apm-20-2134>. (26)

### <https://doi.org/10.1080/22221751.2020.1770129>. (3)

### IL-10/Trauma

### <https://doi.org/10.1155/2015/747036>. (24)

### PMID: [18377749](https://www.ncbi.nlm.nih.gov/pubmed/18377749).

### IL-4/COVID-19

### <https://doi.org/10.1038/s41392-020-0211-1>. (25)

###

### IL-4/Trauma

### <https://doi.org/10.1097/SLA.0000000000001001>. (19)

### WBC/COVID-19

### <https://doi.org/10.1007/s00430-020-00693-z>. (1)

### WBC/Trauma

### <https://doi.org/10.3389/fimmu.2018.00435>. (27)

### <https://doi.org/10.2147/JIR.S101064>. (28)

**Neutrophil/COVID-19**

<https://doi.org/10.1007/s00430-020-00693-z>. (1)

**Neutrophil/Trauma**

<https://doi.org/10.1186/s12974-020-02005-x>. (29)

**Monocyte/COVID-19**

<https://doi.org/10.1007/s00430-020-00693-z>. (1)

**Monocyte/Trauma**

### <https://doi.org/10.2147/JIR.S101064>. (28)

### <https://doi.org/10.3390/ijms18102135>. (30)

### Lymphocyte/COVID-19

<https://doi.org/10.1007/s00430-020-00693-z>. (1)

### <https://doi.org/10.1016/S0140-6736(20)30566-3>. (31)

### Lymphocyte/Trauma

### <https://doi.org/10.1186/s13054-016-1341-2>. (32)

### <https://doi.org/10.3389/fimmu.2019.01501>. (33)

### <https://doi.org/10.3389/fimmu.2018.00435>. (27)

### CD4+ T cell/COVID-19

<https://doi.org/10.3389/fimmu.2020.596631>. (34)

### <https://doi.org/10.1038/s41598-021-81732-4>. (35)

### CD4+ T cell/Trauma

<https://doi.org/10.3389/fimmu.2019.01501>. (33)

### <https://doi.org/10.3389/fimmu.2018.00435>. (27)

**Th1, Th2, Th17, Treg cells/COVID-19**

<https://doi.org/10.1089/vim.2020.0177>. (36)

**Th1, Th2, Th17, Treg cells/Trauma**

PMID: 26885042 (37)

**CD8+ T cell/COVID-19**

<https://doi.org/10.1007/s00430-020-00693-z>. (1)

### <https://doi.org/10.1038/s41598-021-81732-4>. (35)

**CD8+ T cell/Trauma**

<https://doi.org/10.3389/fimmu.2019.01501>. (33)

<https://doi.org/10.1016/j.injury.2014.09.011>. (38)

<https://doi.org/10.1097/00005373-199312000-00007>. (39)

PMID: 3399284 (40)

1. Han M, Xu M, Zhang Y, Liu Z, Li S, He T, et al. Assessing SARS-CoV-2 RNA levels and lymphocyte/T cell counts in COVID-19 patients revealed initial immune status as a major determinant of disease severity. Med. Microbiol. Immunol. 2020;209(6):657-68.

2. Long X, Zhang Z, Zou W, Ling J, Li D, Jing L, et al. Coagulopathy of patients with covid-19 is associated with infectious and inflammatory markers. Risk Manag Healthc Policy. 2020;13:1965-75.

3. Han H, Ma Q, Li C, Liu R, Zhao L, Wang W, et al. Profiling serum cytokines in COVID-19 patients reveals IL-6 and IL-10 are disease severity predictors. Emerg. Microbes Infect. 2020;9(1):1123-30.

4. Giannoudis PV, Smith MR, Evans RT, Bellamy MC, Guillou PJ. Serum CRP and IL-6 levels after trauma: not predictive of septic complications in 31 patients. Acta Orthop. Scand. 1998;69(2):184-8.

5. Li Y, Chen L, Fang W, Chen H. Application value of procalcitonin, Creactive protein and interleukin6 in the evaluation of traumatic shock. Exp. Ther. Med. 2019;17(6):4586-92.

6. Meisner M, Adina H, Schmidt J. Correlation of procalcitonin and C-reactive protein to inflammation, complications, and outcome during the intensive care unit course of multiple-trauma patients. Crit. Care. 2005;10(1):1-10.

7. Nishikura T. Procalcitonin (PCT) production in a thyroidectomized patient. Intensive Care Med. 1999;25(9):1031.

8. Wanner GA, Keel M, Steckholzer U, Beier W, Stocker R, Ertel W. Relationship between procalcitonin plasma levels and severity of injury, sepsis, organ failure, and mortality in injured patients. Crit. Care Med. 2000;28(4):950-7.

9. Sakran JV, Michetti CP, Sheridan MJ, Richmond R, Waked T, Aldaghlas T, et al. The utility of procalcitonin in critically ill trauma patients. J. Trauma Acute Care Surg. 2012;73(2):413-8.

10. Yang G, Hu R, Zhang C, Qian C, Luo Q-Q, Yung W-H, et al. A combination of serum iron, ferritin and transferrin predicts outcome in patients with intracerebral hemorrhage. Sci. Rep. 2016;6(1):1-10.

11. Fu J, Huang P, Zhang S, Yao Q, Han R, Liu H, et al. The value of serum amyloid A for predicting the severity and recovery of COVID19. Exp. Ther. Med. 2020;20(4):3571-7.

12. Carabias CS, Castaño-León AM, Blanca Navarro B, Panero I, Eiriz C, Gómez PA, et al. Serum amyloid A1 as a potential intracranial and extracranial clinical severity biomarker in traumatic brain injury. J. Intensive Care Med. 2020;35(11):1180-95.

13. Wicker E, Benton L, George K, Furlow W, Villapol S. Serum amyloid A protein as a potential biomarker for severity and acute outcome in traumatic brain injury. Biomed. Res. Int. 2019;2019:5967816.

14. Wilfred BS, Madathil SK, Cardiff K, Urankar S, Yang X, Hwang HM, et al. Alterations in Peripheral Organs following Combined Hypoxemia and Hemorrhagic Shock in a Rat Model of Penetrating Ballistic-Like Brain Injury. J. Neurotrauma. 2020;37(4):656-64.

15. Trouillet-Assant S, Viel S, Gaymard A, Pons S, Richard J-C, Perret M, et al. Type I IFN immunoprofiling in COVID-19 patients. J. Allergy Clin. Immunol. 2020;146(1):206-8.

16. Davoudi-Monfared E, Rahmani H, Khalili H, Hajiabdolbaghi M, Salehi M, Abbasian L, et al. A randomized clinical trial of the efficacy and safety of interferon β-1a in treatment of severe COVID-19. Antimicrob. Agents Chemother. 2020;64(9):e01061.

17. Shalhoub S. Interferon beta-1b for COVID-19. Lancet. 2020;395(10238):1670-1.

18. Sallard E, Lescure F-X, Yazdanpanah Y, Mentre F, Peiffer-Smadja N. Type 1 interferons as a potential treatment against COVID-19. Antiviral Res. 2020;178:104791.

19. Namas RA, Vodovotz Y, Almahmoud K, Abdul-Malak O, Zaaqoq A, Namas R, et al. Temporal patterns of circulating inflammation biomarker networks differentiate susceptibility to nosocomial infection following blunt trauma in humans. Ann. Surg. 2016;263(1):191-8.

20. Liu J, Li S, Liu J, Liang B, Wang X, Wang H, et al. Longitudinal characteristics of lymphocyte responses and cytokine profiles in the peripheral blood of SARS-CoV-2 infected patients. EBioMedicine. 2020;55:102763.

21. Heuberger J, Trimpert J, Vladimirova D, Goosmann C, Lin M, Schmuck R, et al. Epithelial response to IFN‐γ promotes SARS‐CoV‐2 infection. EMBO Mol. Med. 2021;13(4):e13191.

22. Rostworowski M, Balasingam V, Chabot S, Owens T, Yong VW. Astrogliosis in the neonatal and adult murine brain post-trauma: elevation of inflammatory cytokines and the lack of requirement for endogenous interferon-γ. Journal of Neuroscience. 1997;17(10):3664-74.

23. Ong EZ, Chan YFZ, Leong WY, Lee NMY, Kalimuddin S, Mohideen SMH, et al. A dynamic immune response shapes COVID-19 progression. Cell Host Microbe. 2020;27(6):879-82.

24. Sousa A, Raposo F, Fonseca S, Valente L, Duarte F, Gonçalves M, et al. Measurement of cytokines and adhesion molecules in the first 72 hours after severe trauma: association with severity and outcome. Dis. Markers. 2015;2015:747036.

25. Xu Z-S, Shu T, Kang L, Wu D, Zhou X, Liao B-W, et al. Temporal profiling of plasma cytokines, chemokines and growth factors from mild, severe and fatal COVID-19 patients. Signal Transduct. Target. Ther. 2020;5(1):1-3.

26. Li J, Rong L, Cui R, Feng J, Jin Y, Yu Y, et al. Dynamic changes in serum IL-6, IL-8, and IL-10 predict the outcome of ICU patients with severe COVID-19. Ann. Palliat. Med. 2021;10(4):3706-14.

27. Serve R, Sturm R, Schimunek L, Störmann P, Heftrig D, Teuben MP, et al. Comparative analysis of the regulatory T cells dynamics in peripheral blood in human and porcine polytrauma. Front. Immunol. 2018;9:435.

28. Bastian OW, Kuijer A, Koenderman L, Stellato RK, van Solinge WW, Leenen LP, et al. Impaired bone healing in multitrauma patients is associated with altered leukocyte kinetics after major trauma. J. Inflamm. Res. 2016;9:69-78.

29. Alam A, Thelin EP, Tajsic T, Khan DZ, Khellaf A, Patani R, et al. Cellular infiltration in traumatic brain injury. Journal of neuroinflammation. 2020;17(1):1-17.

30. Kanazawa M, Ninomiya I, Hatakeyama M, Takahashi T, Shimohata T. Microglia and monocytes/macrophages polarization reveal novel therapeutic mechanism against stroke. International journal of molecular sciences. 2017;18(10):2135.

31. Zhou F, Yu T, Du R, Fan G, Liu Y, Liu Z, et al. Clinical course and risk factors for mortality of adult inpatients with COVID-19 in Wuhan, China: a retrospective cohort study. Lancet. 2020;395(10229):1054-62.

32. Manson J, Cole E, De’Ath HD, Vulliamy P, Meier U, Pennington D, et al. Early changes within the lymphocyte population are associated with the development of multiple organ dysfunction syndrome in trauma patients. Crit. Care. 2016;20(1):1-10.

33. Manson J, Hoffman R, Chen S, Ramadan MH, Billiar TR. Innate-like lymphocytes are immediate participants in the hyper-acute immune response to trauma and hemorrhagic shock. Frontiers in immunology. 2019;10:1501.

34. Peng X, Ouyang J, Isnard S, Lin J, Fombuena B, Zhu B, et al. Sharing CD4+ T cell loss: when COVID-19 and HIV collide on immune system. Front. Immunol. 2020;11:596631.

35. Liu L, Chen Z, Du Y, Gao J, Li J, Deng T, et al. Cd8+ T cells predicted the conversion of common covid-19 to severe. Scientific reports. 2021;11(1):1-11.

36. Sarmiento-Monroy JC, Parra-Medina R, Garavito E, Rojas-Villarraga A. T Helper 17 Response to Severe Acute Respiratory Syndrome Coronavirus 2: A Type of Immune Response with Possible Therapeutic Implications. Viral Immunol. 2021;34(3):190-200.

37. Zhang Y, Li XF, Wu W, Chen Y. Dynamic changes of circulating T-helper cell subsets following severe thoracic trauma. Int. J. Clin. Exp. Med. 2015;8(11):21106.

38. Hua R, Zhang Y, Chen F, Zhou Z, Li X, Shao B, et al. Decreased levels of perforin-positive lymphocytes are associated with posttraumatic complications in patients with major trauma. Injury. 2014;45(12):2089-95.

39. Cheadle WG, Pemberton RM, Robinson D, Livingston DH, Rodriguez JL, Polk Jr HC. Lymphocyte subset responses to trauma and sepsis. J. Trauma. 1993;35(6):844-9.

40. Grob P, Holch M, Fierz W, Glinz W, Geroulanos S. Immunodeficiency after major trauma and selective surgery. Pediatr. Infect. Dis. J. 1988;7(5 Suppl):37-42.
